# Clarification of the clinical significance of an intron variant in a case of Peutz–Jeghers syndrome with abnormal RNA splicing of *STK11*

**DOI:** 10.1186/s13039-025-00710-x

**Published:** 2025-08-20

**Authors:** Aki Ishikawa, Masahiro Gotoh, Mineko Ushiama, Hiromi Sakamoto, Noriko Tanabe, Tomoko Watanabe, Hourin Cho, Masayoshi Yamada, Kokichi Sugano, Kouya Shiraishi, Makoto Hirata, Teruhiko Yoshida, Akihiro Sakurai

**Affiliations:** 1 Department of Medical Genetics and Genomics, School of Medicine, Sapporo Medical University, 1 West 17, Chuo-ku, Sapporo, 060-8556 Japan; 2 Department of Clinical Genomics, National Cancer Center Research Institute, Tokyo, Japan; 3 Department of Genetic Medicine and Services, National Cancer Center Hospital, Tokyo, Japan; 4 Department of Endoscopy, Gastrointestinal Endoscopy Division, National Cancer Center Hospital, Tokyo, Japan; 5 Department of Genetic Counseling, Kyoundo Hospital, Tokyo, Japan

**Keywords:** Peutz–jeghers syndrome, *STK11*, Abnormal splicing, Genetic analysis, Clinical relevance, U12-type intron

## Abstract

Peutz–Jeghers syndrome is an autosomal dominant disease characterized by intestinal polyposis, mucocutaneous pigmentation, and an increased risk of various types of cancer. Germline mutations in *STK11* (*LKB1*), which encodes serine/threonine kinase 11, have been identified as the major cause of Peutz–Jeghers syndrome. Here, we detected a rare variant of undetermined significance in intron 2 of *STK11* using multi-gene panel analysis in a girl with clinically suspected Peutz–Jeghers syndrome. We confirmed this variant caused abnormal splicing in exons 2 and 3 using reverse transcription-PCR and Sanger sequencing. To validate the predicted impact of this variant on splicing, we performed functional analysis using a minigene assay. Functional analysis experiments demonstrated that this variant suppresses normal splicing, and the clinical significance of the STK11 variant, which was initially considered a variant of “uncertain significance,” was reclassified as “likely pathogenic” based on functional analysis. The interpretation of U12-type intronic variants remains particularly challenging due to limited data and the absence of specific recommendations in existing guidelines [1, 2], and when segregation analysis is difficult, functional analysis from splicing assays is essential to provide accurate genetic diagnosis and information for clinical management. However, further familial segregation analysis and further validation of potential exon-skipping events are necessary to fully characterize the splicing impact of this variant.

**Supplementary Information:**

The online version contains supplementary material available at 10.1186/s13039-025-00710-x.

## Introduction

Peutz–Jeghers syndrome (PJS; OMIM #175200) is a rare autosomal dominant disorder characterized by mucocutaneous pigmentation, gastrointestinal hamartomatous polyposis, and a high risk of various neoplasms [3, 4]. Germline mutations in *STK11* (OMIM #602216), which encodes serine/threonine kinase 11, a tumor suppressor that has roles in apoptosis, cell cycle arrest, cell proliferation, cell polarity, and energy metabolism, have been identified as the sole cause of PJS [5, 6]. *STK11* is located on human chromosome 19p13 with nine coding exons and one non-coding exon and spans approximately 25 kb [7]. *STK11* haploinsufficiency contributes to the pathogenesis of PJS [8–10]. More than 300 pathogenic variants of *STK11* have been reported in individuals with PJS. All types of variants have been reported, from missense variants to whole-gene deletion. RNA splicing abnormalities in the minor U12-type intron 2 of *STK11* may also lead to the development of PJS [11].

Minor U12-type introns account for approximately 0.3% of all introns and contain conserved sequences essential for cell survival and homeostasis. Mutations in components of the U12 spliceosome are associated with congenital or somatic disorders, including PJS [12–14]. Unlike U2-type introns, U12-type introns are extremely rare. Data on the pathogenicity of U12-type intron variants are limited and they are particularly challenging because of the lack of specific recommendations in existing guidelines [1], including the 2023 Splicing-Specific Mutation Interpretation (SVI) guidelines [2]. While the pathogenicity of U12-type intron variants remains challenging to assess, functional analysis plays a crucial role in complementing clinical evaluation, particularly in the absence of segregation data.

In this study, we characterized a single nucleotide variant in intron 2 of *STK11* detected in a case with clinically suspected PJS by demonstrating RNA splicing suppression using a minigene assay.

## Materials and methods

### Clinical samples and germline genetic testing

A multi-gene panel test (NCC Oncopanel for Familial Cancers [NOP_FC] ver. 3.0), which covered 147 hereditary cancer-related genes, was performed using genomic DNA derived from a peripheral blood sample from the proband. For this multi-gene panel testing, we used SureSelect Custom DNA Target Enrichment Probes (Agilent, Santa Clara, CA), which were originally designed to target 147 cancer-predisposing genes, including APC (adenomatous polyposis coli) and other genes associated with colorectal cancer or polyposis. The target regions cover exons, intronic sequences at the exon-intron junctions (approximately 25 bp), promoters, and regulatory regions within the introns of high-penetrance genes. Subsequently, sequencing and data analysis was performed using NextSeq (Illumina, San Diego, CA) and csDAI ver3.0 (Mizuho Res. & Technol., Tokyo, Japan).

For analysis of STK11 transcripts, cDNAs were synthesized with oligo (dT)20 and SuperScript IV Reverse Transcriptase (Thermo Fisher Scientific, Waltham, MA) using RNAs derived from peripheral blood samples from the proband and normal controls. Prior to RNA extraction, half of the blood samples were treated with puromycin to inhibit nonsense-mediated mRNA decay. Puromycin (Merck, Darmstadt, Germany) was added to the samples to a final concentration of 0.2 mg/ml to the samples and incubated at 37 °C for 3 h, according to the previous report [15]. Then, PCR and Sanger sequencing were performed using AmpliTaq Gold DNA Polymerase (Thermo Fisher Scientific). PCR primers are listed in Supplementary Table 1.

### Cell lines

The IMR-90 (ATCC CCL-186) normal human fibroblast cell line and HCT116 (RCB2978) human colorectal cancer cell line were provided by the American Type Culture Collection and RIKEN BRC through the National Bio-Resource Project of MEXT/AMED, Japan, respectively. The cell lines were maintained under standard conditions according to the guidelines provided by ATCC (CCL-186) and RIKEN BRC (RCB2987).

### Plasmid construction

For reverse transcription (RT)-PCR using a minigene assay, a 1.1-kbp fragment containing exon 2 to exon 3 of *STK11* (NC_000019.10:g.1,218,361–1,219,495) was amplified from RNA of IMR-90 cells. The DNA fragment containing the wild-type (WT) sequence was subsequently cloned into the pcDNA3 vector (Invitrogen, Carlsbad, CA). Vector DNA was mutated by site-directed mutagenesis to introduce the variant NM_000455.5:c.375–10 A > G. PCR primers are listed in Supplementary Table 2.

For a luciferase reporter assay, a 823-bp fragment containing WT or mutated (mut) intron 2 of *STK11* (NC_000019.10:g.1,218,501–1,219,323) was subsequently cloned and inserted into the NanoLuciferase (Nluc) region of the CMV promoter-driven pNL vector (Promega, Madison, WI) by site-directed mutagenesis, and new 5′ and 3′ splice sites were introduced into the vector construct by these intron insertions. PCR primers are listed in Supplementary Table 3.

### Minigene assay

The HCT116 colorectal cancer cell line was transiently transfected with the pcDNA3-derived expression vectors pSTK11-wt or pSTK11-mt. RT-PCR was performed with vector-specific primers using RNA extracted at 24 h after transfection. PCR primers are listed in Supplementary Table 4.

The HCT116 cell line was transiently transfected with the pNL-derived expression vectors pSTK11-wt-Nluc or pSTK11-mt-Nluc. Simultaneously, they were co-transfected with pGL4(firefly luciferase/CMV) (Promega) as a control vector. Luciferase activity in three independent experiments was measured at 24 h after transfection using the Nano-Glo Dual-Luciferase Reporter Assay System (Promega). Firefly luciferase activity was normalized to that of Nluc.

## Results

### Clinical data

**Fig. 1:**
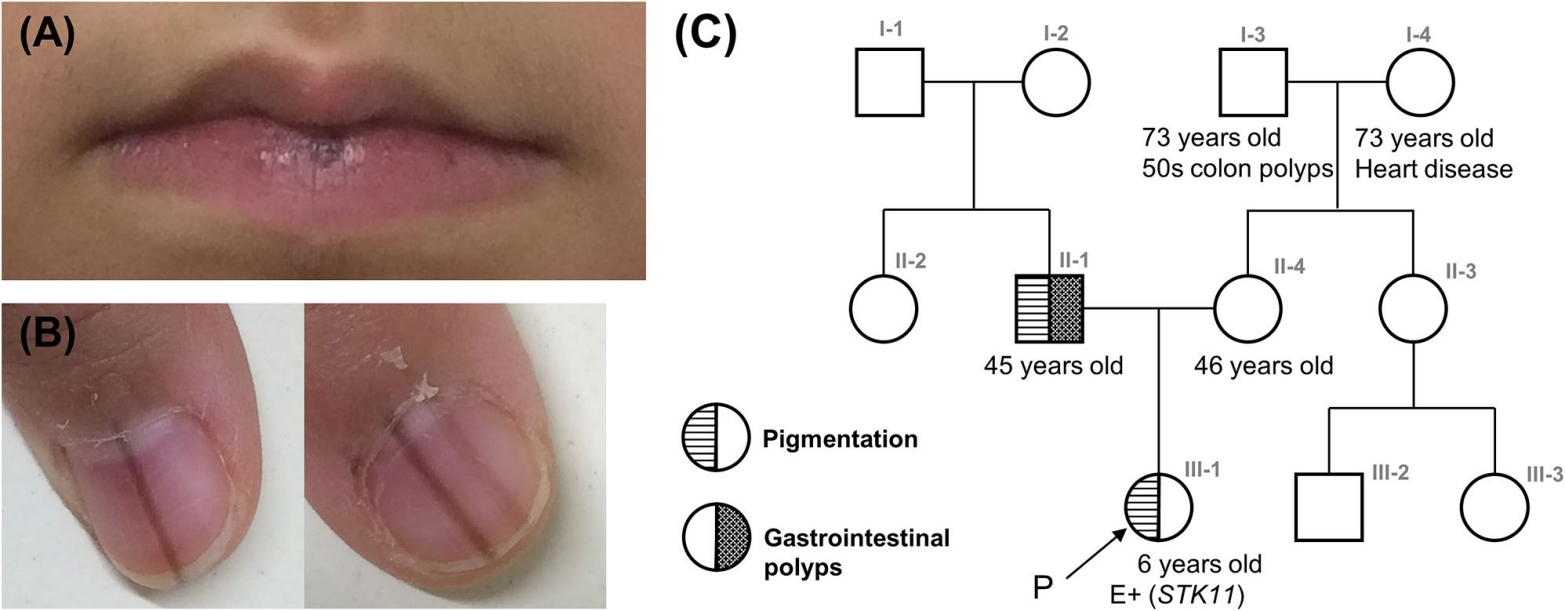
Clinical photograph of the patients and pedigree. **(A**,** B)** Clinical findings of the proband. **(A)** Note the black pigment spots on the lips. **(B)** Linear pigmentation of the nail plate on the right index finger and left thumb. **(C)** Pedigree of the patients with clinically suspected Peutz–Jeghers syndrome

The patient was a 6-year-old girl with no siblings, born at term with a normal delivery. She had black pigmentation on the gingiva and upper eyelid mucosa from the age of 1 year 6 months and multiple nevi on her lips at the age of 2 years (Fig. 1A). At the age of 4 years, pigment striations appeared on the nails of both her thumb nails and on the second and fourth toes of her right foot (Fig. 1B). She had constipation at the age of 4 years, but her bowel movements were well controlled with medication.

Regarding her family history, her father (age 45 years) had multiple lentigines on his lips, oral cavity, fingers, and pubic area, and had undergone multiple colonic polypectomies. He also had a history of treatment for gastric ulcers, hematemesis, and gastrointestinal polyps when he was in his 20s. The pathology of the polyps and other information are unknown. The patient’s mother (age 46 years) had a history of vascular embolization for left renal angiomyolipoma at the age of 35 years. She also had a history of uterine fibroids, endometriosis, and a neuroma on the left lower extremity, but had no other findings suggestive of tuberous sclerosis or neurofibromatosis type 1 (Fig. 1C).

According to the NCCN Guidelines Version 3.2024 for Peutz-Jeghers Syndrome [16], a clinical diagnosis requires the presence of two or more of the following features: (1) two or more Peutz-Jeghers-type hamartomatous polyps of the gastrointestinal tract, (2) mucocutaneous hyperpigmentation of the mouth, lips, nose, eyes, genitalia, or fingers, and (3) a family history of PJS. Although the patient exhibited mucocutaneous pigmentation and had a family history of gastrointestinal findings suggestive of PJS, she did not fully meet the diagnostic criteria at this time because Peutz-Jeghers-type hamartomatous polyps were not identified. Genetic testing was performed at the age of 6 years to confirm the diagnosis.

### Detection of STK11 germline mutations

**Fig. 2:**
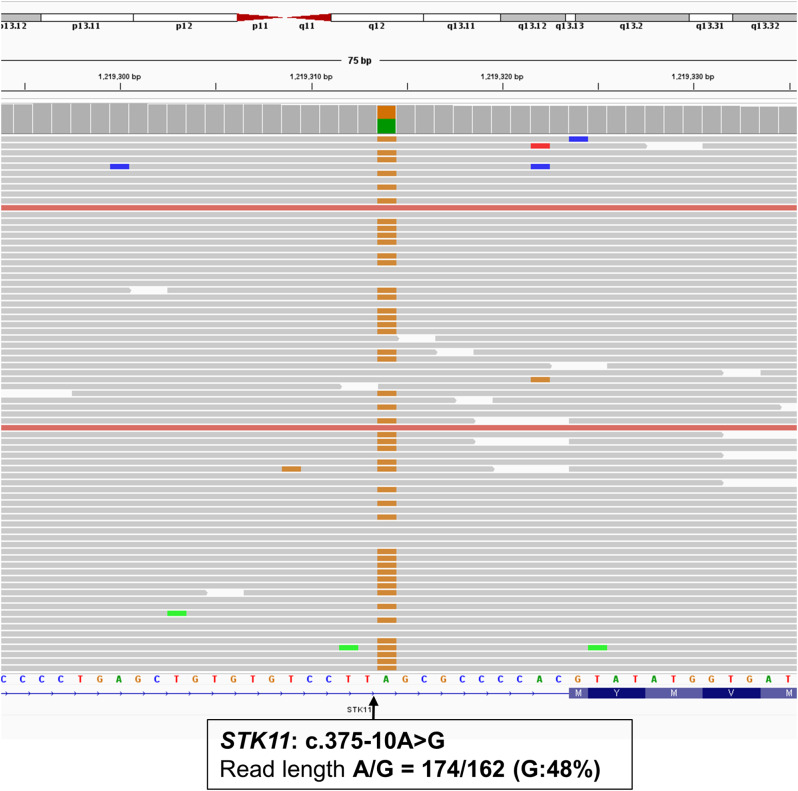
Detection of an intron variant in the STK11 by NCC Oncopanel for Familial Cancers (ver. 3.0). DNA derived from the peripheral blood cells of the proband was used to perform multi-gene panel analysis with the NCC Oncopanel for Familial Cancers (ver. 3.0). *STK11*: NM_000455.5:c.375–10 A > G was detected as a single allele (48%)

DNA derived from peripheral blood cells of the proband was used to perform multi-gene panel analysis with the NOP_FC. As clinical research, NOP_FC analysis (ver. 3.0) was performed to investigate the variants detected in the csDAI 3 mutation call. Of the variants detected in the entire coding region and exon–intron junctions (approximately 25 bp), two single nucleotide variants with an alternative allele frequency of less than 0.005 based on the gnomAD population database and found in genes associated with hereditary tumors corresponding to the clinical diagnosis of the proband were detected: *STK11*: NM_000455.5:c.375–10 A > G: intron 2: heterozygote, the allele frequency for this variant was 0.00 in both the gnomAD v4.1.0 and JMorp 38 K databases, which was classified as “Uncertain Significance ”(PM2) according to the American College of Medical Genetics and Genomics (ACMG)/Association for Molecular Pathology (AMP) 2015 variant interpretation guidelines [15]; and *STK11*: NM_000455.5:c.735-38G > C: intron 5: heterozygote, which was classified as “Likely Benign” (BP4, BP7). These variants have not been validated by Sanger sequencing. The intron variant of Uncertain Significance (*STK11*: NM_000455.5:c.375-10A > G) was detected as a single allele (48%) (Fig. 2).

Other analyses included a multiplex ligation–dependent probe amplification assay of *STK11* (SALSA P101-B4 MLPA KIT; MRC-Holland, Amsterdam, Netherlands), which showed no copy number changes in either probe.

### RT-PCR-Sanger sequencing detection of abnormal RNA splicing of STK11 intron 2

**Fig. 3:**
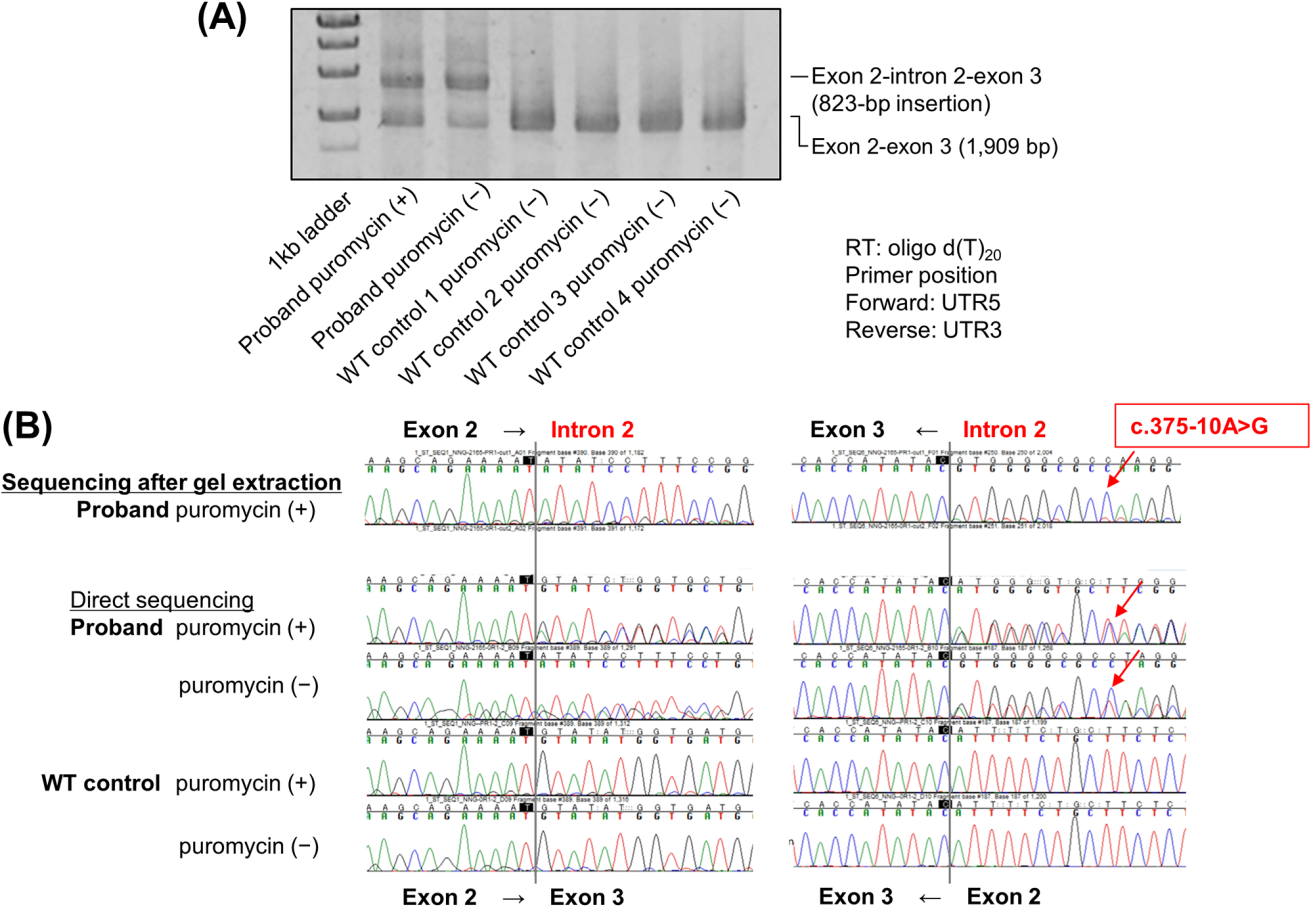
Detection of RNA splicing abnormalities by RT-PCR and validation of RNA splicing abnormalities by Sanger sequencing. **(A)** Reverse transcription (RT)-PCR products encompassing *STK11* exons 2–3 were analyzed. **(B)** Sanger sequencing at both ends of the cut fragments showed that intron 2 was included. WT, wild-type

RT-PCR and Sanger sequencing were performed using RNA derived from the peripheral blood cells of the proband. In addition to the normal exon 2-exon 3 splicing-derived PCR product found in WT control samples, a splicing-derived PCR product retaining intron 2 (823 bp) was identified, corresponding to r.374_375ins374 + 1_375-1. Furthermore, the single nucleotide variant c.375–10 A > G was only detected in PCR products retaining intron 2. These data suggest that an RNA splicing error could occur in the variant allele, resulting in a frameshift mutation leading to p.(Met125Ilefs*140) (Fig. 3).

### Verification of the RNA splicing inhibitory effect of the variant

**Fig. 4:**
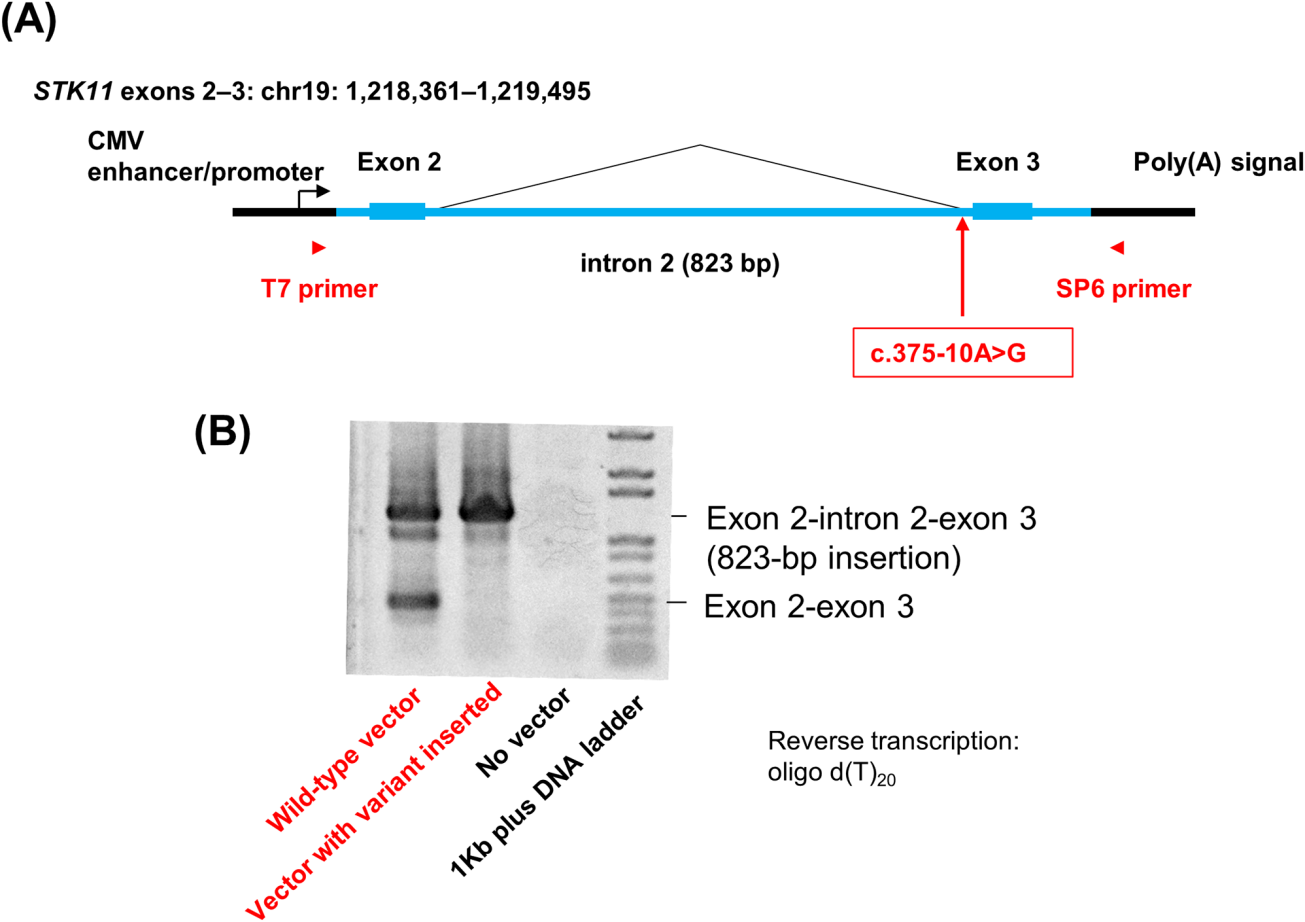
*STK11* minigene assay vector (**A**) and verification of the RNA splicing inhibitory effect of the variant by reverse transcription-PCR (qualitative experiment) (**B**)

A minigene assay using expression vectors demonstrated that RNA splicing around exons 2 and 3 of *STK11* could be reproduced in the HCT116 human colorectal cancer cell line. Vector-specific primers were used to exclude endogenous *STK11* expression in RT-PCR analysis. PCR products derived from normal RNA splicing were lost, while those from intron 2 retention were found in the variant vector sample. This result showed that normal RNA splicing was completely suppressed by the single nucleotide variant c.375–10 A > G. While both PCR products were detected in the WT vector sample, which would be influenced by the low efficiency of minor splicing compared with that of the CMV promoter in this minigene assay (Fig. 4).

### Verification of the RNA splicing suppression effect of the variant

**Fig. 5:**
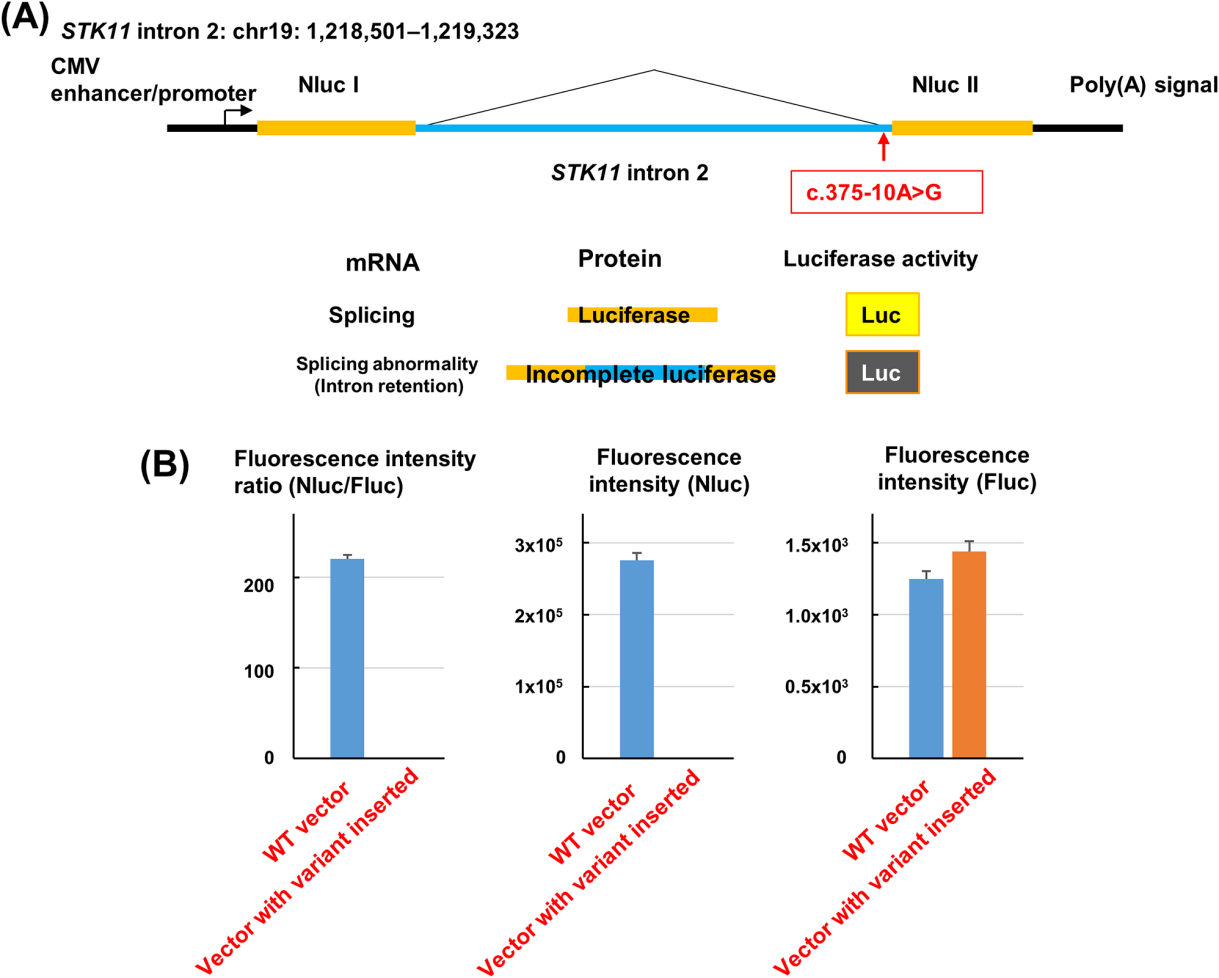
*STK11* minigene assay NanoLuciferase (Nluc) vector (**A**) and verification of the RNA splicing suppression effect of the variant using a reporter assay (quantitative experiment) (**B**). Fluc, firefly luciferase; WT, wild-type

An Nluc expression vector containing the *STK11* intron 2 region was created, and a variant insertion vector was created using mutagenesis. The WT vector showed fluorescence associated with normal splicing, while the fluorescence produced by the variant insertion vector was significantly reduced compared to the WT vector. The loss of fluorescence due to the suppression of normal RNA splicing was confirmed in the variant insertion vector (Fig. 5). The details of the Luciferase reporter assay are shown in Supplementary Table 5. These results confirmed that the *STK11* variant, initially thought to be a variant of Uncertain Significance, suppressed normal splicing, and in accordance with the ACMG/AMP guidelines [1], the evaluation of PS3 was added, and its clinical significance was changed to Likely Pathogenic (PS3, PM2).

## Discussion

Minor U12-type introns account for only about 0.3% of all introns, but they have evolutionarily conserved sequences and play an important role in cell survival and homeostasis. In recent years, their association with splicing abnormalities and disease has become apparent. Reports have suggested that splicing abnormalities in the minor U12-type intron of the tumor suppressor gene STK11, which is the causative gene for PJS, may lead to the development of PJS [11]. In this study, we demonstrated that the STK11 c.375–10 A > G variant leads to intron 2 retention (r.374_375ins374 + 1_375-1), resulting in a frameshift mutation (p.(Met125Ilefs*140)). Functional analyses, including a minigene assay and a luciferase reporter assay, indicated that this variant significantly suppressed normal RNA splicing. These findings contribute to the growing understanding of U12-type intron variants in STK11-related disorders.

Despite the functional evidence provided in this study, several limitations remain. One major limitation is the inability to perform familial segregation analysis. Although the patient’s father exhibited features suggestive of PJS, genetic testing was not feasible due to family circumstances. Segregation analysis could have provided additional evidence supporting the pathogenicity of the variant, particularly in determining whether it co-segregates with the disease. Additionally, further validation of potential exon-skipping events is necessary to fully characterize the splicing impact of this variant.

Another challenge in interpreting U12-type intron variants is the lack of specific recommendations in existing variant classification guidelines. While the 2023 Splicing-Specific Variant Interpretation (SVI) guidelines [2] provide a framework for evaluating splicing variants, they do not specifically address the unique characteristics of U12-type introns. This indicates the need for further refinement of classification criteria to ensure accurate interpretation of these rare intronic variants.

Expanding publicly available datasets through variant submissions to repositories such as ClinVar is essential for improving the collective understanding of U12-type intron variants. Given the rarity of these variants, accumulating data from multiple studies will aid in establishing their clinical significance. The inclusion of functional data in variant databases will also facilitate more robust classification frameworks in the future.

Future studies are necessary to further elucidate the pathogenic mechanisms of STK11 intronic variants. In vivo functional analyses, such as the development of animal models, could provide deeper insights into the physiological impact of these variants. Additionally, alternative splicing events, such as potential exon skipping, should be explored in greater detail using targeted experimental approaches. These studies will be crucial for refining variant classification strategies and improving diagnostic accuracy for patients with suspected PJS.

In summary, our study provides new insights into the impact of the STK11 c.375–10 A > G variant on RNA splicing. However, further familial segregation analysis and further validation of potential exon skipping events are needed to fully clarify the impact of this variant on splicing. And the interpretation of U12 intronic variants remains challenging due to limited data and lack of specific guidelines. Further studies, including familial segregation studies and in vivo functional analysis, will be essential for comprehensive variant classification.

## Electronic supplementary material

Below is the link to the electronic supplementary material.

Supplementary Material 1

Supplementary Material 2

## Data Availability

No datasets were generated or analysed during the current study.
