# Supplementary material for: Clarification of the clinical significance of an intron variant in a case of Peutz–Jeghers syndrome with abnormal RNA splicing of STK11
Source: Mol Cytogenet. 2025 Aug 20;18:20. doi: 10.1186/s13039-025-00710-x (PMC12366057; doi:10.1186/s13039-025-00710-x)
Supplement: Supplementary file 2 — Supplementary Material 2 [file 13039_2025_710_MOESM2_ESM.docx]

Supplementary Information for

**Clarification of the clinical significance of an intron variant in a case of Peutz–Jeghers syndrome with abnormal RNA splicing of *STK11***

Aki Ishikawa, Masahiro Gotoh, Mineko Ushiama, Hiromi Sakamoto, Noriko Tanabe, Tomoko Watanabe, Hourin Cho, Masayoshi Yamada, Kokichi Sugano, Kouya Shiraishi, Makoto Hirata, Teruhiko Yoshida, Akihiro Sakurai

**Contents**

Supplementary Table 1

Supplementary Table 2

Supplementary Table 3

Supplementary Table 4

Supplementary Table 5

Supplementary Table 1. Primers for reverse transcription-PCR

| Primer | Sequence (5′–3′) | Position (NM_000455.5) | Size of the normal amplicon |
| --- | --- | --- | --- |
| ST_rtF | ACTGCGCTCGGCCGTGTTCATACTTGTCC | c.-422_-394 (Exon 1) | 1,909 bases |
| ST_rtR | GCTGCGCCCGGTCCCTGCTGTC | c.*185_*164 (Exon 10) | - |

Supplementary Table 2. Primers for vector construction and site-directed mutagenesis

| Primer | Sequence (5′–3′) |
| --- | --- |
| STK11_IVS1_F1 | CAGGCCATCATCCTGACGTTGG |
| STK11_IVS3_R1 | GCAGTGTGGCCTCACGGAAA |
| STK11_IVS2_mR1 | CAAGGACACACAGCTCAGGG |
| STK11_IVS2-e3_F1 | GCGCCCCACGTATATGGTG |

Supplementary Table 3. Primers for vector construction and site-directed mutagenesis

| Primer | Sequence (5′–3′) |
| --- | --- |
| Nluc_R4 | CTTAAAGTGATGATCATCCACAGGG |
| STK11_IVS2_F2 | ATATCCTTTCCGGTGTTGGGAC |
| STK11_IVS2_R2 | GTGGGGCGCTAAGGACACAC |
| STK11_IVS2_mR2 | GTGGGGCGCCAAGGACAC |
| Nluc_F4-3 | GTGATCCTGCACTATGGCACACT |

Supplementary Table 4. Primers for vector-specific reverse transcription-PCR

| Primer | Sequence (5′–3′) |
| --- | --- |
| T7_20 | TAATACGACTCACTATAGGG |
| SP6_20 | GCATTTAGGTGACACTATAG |

Supplementary Table 5. Luciferase reporter assay

| WT | 1 | 2 | 3 | Average | SD |
| --- | --- | --- | --- | --- | --- |
| Nluc | 65916 | 69746 | 70863 |  |  |
| Fluc | 300 | 310 | 327 |  |  |
| Ratio | 219.7 | 225.0 | 216.7 | 220.5 | 4.2 |
| Mut | 1 | 2 | 3 | Average | SD |
| Nluc | 82 | 60 | 63 |  |  |
| Fluc | 1363 | 1446 | 1506 |  |  |
| Ratio | 0.0602 | 0.0415 | 0.0418 | 0.0478 | 0.0107 |
| -test | *P*=8.7E-08 |  |  |  |  |

Fluc, firefly luciferase; Mut, mutant; Nluc, NanoLuciferase; SD, standard deviation; WT, wild type.
